# Direct Initiation of Levodopa–Carbidopa Intestinal Gel Infusion After a Positive Levodopa Challenge Test in Advanced Parkinson's Disease

**DOI:** 10.1002/brb3.70193

**Published:** 2024-12-11

**Authors:** Vili Viljaharju, Tuomas Mertsalmi, K. Amande M. Pauls, Maija Koivu, Johanna Eerola‐Rautio, Marianne Udd, Eero Pekkonen

**Affiliations:** ^1^ Department of Neurology Helsinki University Hospital Helsinki Finland; ^2^ Department of Clinical Neurosciences (Neurology) University of Helsinki Helsinki Finland; ^3^ Department of Gastroenterological Surgery Helsinki University Hospital Helsinki Finland

## Abstract

**Introduction:**

Levodopa–carbidopa intestinal gel (LCIG) is an established treatment option in advanced Parkinson's disease (PD). LCIG treatment is usually initiated with a nasojejunal tube (NJT) test phase before percutaneous endoscopic transgastric jejunostomy (PEG‐J) tube installation. However, some centers have used direct initiation with PEG‐J. Data comparing these approaches are scarce. The objective of this study was to analyze the risks and benefits of direct PEG‐J initiation after a positive levodopa challenge test (LCT) for selected patients compared to initiation with a temporary NJT test phase.

**Methods:**

Thirty‐three consecutive advanced PD patients commenced LCIG‐treatment between February 2016 and December 2019 at Helsinki University Hospital. Of them, 11 (33%) selected patients had direct initiation without an NJT test phase. Treatment discontinuations and adverse events during the first 6 months of treatment were evaluated retrospectively. The duration of hospital stay related to the initiation of the treatment was compared between the groups.

**Results:**

Between the direct initiation and NJT test phase groups, there were no significant differences in treatment discontinuations (0 vs. 1, respectively); the number of inner tube or PEG‐J tube replacements (1 vs. 3); or infection complications (1 vs. 3) during the first 6 months of treatment. Direct initiation significantly reduced the hospital stay related to treatment initiation (mean 7 vs. 9 days, *p* = 0.001).

**Conclusion:**

For selected patients, the direct initiation of LCIG after a positive LCT, without a temporary NJT test phase, appears safe and does not lead to additional treatment discontinuations or complications.

## Graphical abstract

For selected patients, the direct initiation of levodopa–carbidopa intestinal gel treatment after a positive levodopa challenge test, without a temporary nasojejunal tube test phase, appears safe and does not lead to additional treatment discontinuations or complications.

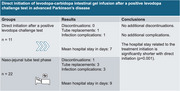

## 1 Introduction

Levodopa is the most effective treatment to alleviate the motor symptoms of Parkinson's disease (PD). (Olanow, Stern, and Sethi 2009) However, the progression of the disease and long‐term medication lead to motor fluctuations and dyskinesia typically after 5–10 years of levodopa treatment (Fox and Lang 2008). Along with other device‐assisted therapies, namely, deep brain stimulation (DBS) and apomorphine infusion, levodopa–carbidopa intestinal gel (LCIG) is an effective treatment to reduce motor fluctuations and dyskinesia in advanced PD (Wirdefeldt, Odin, and Nyholm 2016). LCIG has also been shown to alleviate non‐motor symptoms (NMS) (Honig et al. 2009), the freezing of gait phenomenon (Zibetti et al. 2018), and enhance quality of life (Ciurleo et al. 2018).

The levodopa challenge test (LCT) is widely used as part of the preoperative DBS evaluation to confirm levodopa responsiveness and suitability for DBS treatment (Bronstein et al. 2011). In addition, a positive LCT is a strong predictive factor for a good response to DBS treatment (Kleiner‐Fisman et al. 2006). In the LCT, the patient is assessed in OFF‐state after one night's withdrawal of antiparkinsonian medication and in ON‐state approximately an hour after the patient has received 1.5 times the normal morning levodopa dose in immediate‐release formulation. The Unified Parkinson's Disease Rating Scale III (UPDRSIII) score is measured in both states, and a decrease of more than 30% in the UPDRSIII score is considered to indicate levodopa responsiveness (Saranza and Lang 2021).

In many centers, levodopa responsiveness, suitability, and titration of LCIG treatment, before percutaneous endoscopic transgastric jejunostomy (PEG‐J) tube installation, is confirmed by levodopa–carbidopa infusion via temporary nasojejunal tube (NJT) (Antonini et al. 2017; Buongiorno et al. 2015; Nyholm, Klangemo, and Johansson 2012; Pickut et al. 2014). However, NJT may cause discomfort to patients, is time consuming, and susceptible to complications (Blumenstein 2014). Consequently, some centers have also used direct PEG‐J tube initiation (Honig et al. 2009; Moes et al. 2020; Standaert et al. 2017). Yet, data regarding the role of the NJT test phase, and the risks and benefits of direct PEG‐J initiation, are scarce.

We piloted direct PEG‐J initiation after a positive LCT in selected patients at our movement disorder unit at Helsinki University Hospital between the years 2016 and 2019. In this retrospective observational cohort study, we investigated the risks and benefits of direct PEG‐J initiation after a positive LCT.

## 2 Materials and Methods

In this retrospective, longitudinal observational study, 33 consecutive advanced PD patients commenced LCIG‐treatment between February 2016 and December 2019 at Helsinki University Hospital. Of them, 11 (33%) had direct initiation without an NJT test phase. Levodopa responsiveness was ensured by an antecedent LCT. The patients were not randomized; rather, the choice between direct initiation and an NJT trial was made on a case‐by‐case basis at the discretion of the movement disorder specialists of our unit. Mandatory reasons for a prior NJT trial were cognitive decline and living alone. If a patient had experienced major neuropsychiatric issues, such as hallucinations, in the past, an NJT trial was preferred. An NJT trial was also recommended if either the patient or caregiver faced challenges in learning to use the device. Apart from neuropsychiatric and cognitive symptoms, other motor symptom or NMS did not influence patient selection. Treatment assignments were determined by movement disorder specialists based on individual evaluations, and patients did not have the option to choose whether to start with NJT or not.

Levodopa responsiveness was evaluated with LCT for all patients. For patients with direct initiation, the patients were assessed in OFF‐state after overnight withdrawal of antiparkinsonian medication and in ON‐state 1–2 h after receiving 1.5 times their normal morning levodopa dose in immediate‐release formulation. For patients with temporary NJT, ON‐state assessment was done after the infusion had been on for 1–2 h. A reduction of > 30% in the UPDRSIII score was required to initiate the LCIG treatment via PEG‐J tube.

Inclusion criteria were advanced PD with motor fluctuations and/or disabling dyskinesia poorly controlled with oral medication. Exclusion criteria were advanced dementia, which is defined as a Mini Mental State Examination (MMSE) score < 20 in our center (Folstein et al. 1975), clinical suspicion of atypical parkinsonism, insufficient responsiveness to levodopa, and ongoing psychosis unresponsive to antipsychotic medication. Our clinical protocol is thoroughly described in a recently published article (Viljaharju et al. 2022).

Patients were evaluated as part of our center's routine practice before LCIG initiation and at 6 months during the treatment. All data were extracted from the electronic records of Helsinki University Hospital.

### 2.1 Clinical Data

All information was collected from the patient records including the following baseline data: age, gender, clinical state of PD evaluated by the Hoehn and Yahr score (H&Y), MMSE, living situation, medication, and duration of PD (from the date of diagnosis to LCIG initiation). If not directly stated in patient records, the H&Y was evaluated from the data available.

The duration of the hospital stay related to treatment initiation, complications and early treatment discontinuations were collected from follow‐up data.

UPDRSIII score (OFF and ON) was measured during the LCT before treatment initiation and the UPDRSIII score (infusion ON) was controlled at 6 months in outpatient clinic settings reflecting the patient's everyday response to LCIG.

### 2.2 Statistics

Statistical calculations were generated using IBM SPSS Statistics 27 (International Business Machines Corporation, Endicott, NY). To analyze differences in the hospital stay between patients with temporary NJT and direct initiations, the Mann–Whitney test was used. To compare the means of age, disease duration, and MMSE at baseline, an independent samples *t*‐test was used. For correlation analysis between the UPDRSIII ON score at baseline and the UPDRSIII ON score at 6 months, Pearson correlation analysis was used. All *p* values reported are two‐tailed and a *p* ≤ 0.05 was considered statistically significant.

### 2.3 Objectives

We analyzed the risks and benefits of direct PEG‐J initiation after a positive LCT for selected patients compared to initiation following a temporary NJT test phase.

## 3 Results

Thirty‐three consecutive advanced PD patients commenced LCIG treatment. Patients' epidemiological data are shown in Table 1.

**TABLE 1:** Epidemiological data.

	Direct initiation	NJT test	*p*
*N*	11	22	
Gender (female), *n* (%)	7 (64)	12 (55)	
Age at intervention (years), mean (SD)	73 (5.3)	70 (9.2)	0.267
Disease duration (years), mean (SD)	11 (4.3)	12 (5.1)	0.618
HY stage, mean (SD)	3.4 (0.8)	3.5 (0.7)	
HY stage, *n* (%)			
1	0	0	
2	2 (18)	2 (9)	
3	3 (27)	8 (36)	
4	6 (55)	11 (50)	
5	0	1 (5)	
MMSE, mean (SD)	28 (2.6)	27 (2.8)	0.367
Living situation, *n* (%)			
Alone	0	9 (41)	
Spouse	11 (100)	11 (50)	
Institution	0	2 (9)	
Medication, *n* (%)			
Memory	0	3 (14)	
Antidepressant	1 (9)	8 (36)	
Antipsychotic	1 (9)	5 (23)	

*Note*: Baseline features of the 33 study patients.

Abbreviations: HY, Hoehn and Yahr index; MMSE, Mini‐Mental State Examination; NJT, nasojejunal tube; SD, standard deviation.

Between the direct initiation and the NJT test phase groups, there were no significant differences in treatment discontinuations (0 vs. 1, respectively); the number of inner tube or PEG‐J tube replacements (1 vs. 3); or infection complications (1 vs. 3) during the first 6 months of treatment.

The direct initiation of LCIG after a positive LCT without a temporary NJT trial significantly reduced hospital stay related to treatment initiation (mean 7 vs. 9 days, *p* = 0.001).

The LCT ON phase UPDRSIII score showed a significant positive correlation with the UPDRSIII ON score at 6 months in outpatient clinic settings (*r*(20) = 0.70, *p* = 0.001).

## 4 Discussion

Traditionally LCIG treatment has been initiated with a temporary NJT trial before PEG‐J installation to verify efficacy and suitability; and optimise the drug dose (Antonini et al. 2017; Buongiorno et al. 2015; Nyholm, Klangemo, and Johansson 2012; Pickut et al. 2014). The present study suggests that, for selected patients, direct initiation after a positive LCT, is safe and does not lead to additional treatment failures or complications compared to initiation with an NJT trial. Furthermore, hospital stays with direct initiation are significantly shorter than with an NJT trial. However, these findings may be related to better cognitive function and fewer neuropsychiatric problems in the selected direct initiation patients. The use of memory, antidepressant, and antipsychotic medication was more prevalent in the NJT test group.

Because living alone appears to be a risk factor for early discontinuation of LCIG treatment (Viljaharju et al. 2022), we chose direct initiation only for patients with a caregiver. Similarly, because lower cognitive function and neuropsychiatric problems pose risks to tube dislocations and thus to discontinuation of the treatment (Viljaharju et al. 2022; Devos 2009), we chose to initiate the treatment of these patients with a temporary NJT test phase. However, other motor symptom or NMS, apart from cognitive and neuropsychiatric ones, did not affect patient selection.

The findings of the present study are comparable with earlier reports that have shown that direct initiation can be done safely (Standaert et al. 2017; Olanow et al. 2014). Yet, these studies did not perform comparisons to initiation with an NJT.

In earlier studies, discontinuation during the NJT test phase appears rare but not negligible. For example, Fernandez et al. (2015) reported that of 354 non‐demented patients undergoing NJT evaluation, 5 discontinued because of lack of efficacy, 5 because of adverse effects and 12 withdrew consent (Fernandez et al. 2015).

In comparison, in an older and more cognitively affected patient group, where the prevalence of PD dementia was 50%, lack of efficacy in the NJT test phase was detected in 5% of the patients (Devos 2009). Following these findings, in the present study, similar to DBS evaluation, the LCT was used to ensure levodopa responsiveness before initiation of LCIG treatment. The LCT ON phase UPDRSIII score showed a significant positive correlation with the UPDRSIII ON score at 6 months in outpatient clinic settings questioning the need for an NJT test phase in evaluating treatment effectiveness.

## 5 Conclusion

The present study has some limitations. The patient groups were selected, which makes, for example, the interpretation of differences in hospital stay difficult. Also, the retrospective nature of the study results in some missing data.

In conclusion, for selected patients, the direct initiation of LCIG after a positive LCT, without a temporary NJT test phase, appears safe and does not lead to additional treatment discontinuations or complications. In the future, studies broadening the inclusion criteria for direct initiation would be valuable since patients living alone or with significant cognitive decline were excluded from the direct initiation group in the present study.

## Author Contributions

**Vili Viljaharju**: writing–review & editing, writing–original draft, investigation, formal analysis, visualization, methodology, funding acquisition. **Tuomas Mertsalmi**: methodology, investigation formal analysis, writing–original draft, writing–review & editing. **K. Amande M. Pauls**: conceptualization, methodology, validation, supervision, resources. **Maija Koivu**: conceptualization, methodology, validation supervision, resources. **Johanna Eerola‐Rautio**: conceptualization, methodology, supervision, resources, validation. **Marianne Udd**: conceptualization, methodology, validation, supervision, resources. **Eero Pekkonen**: conceptualization, methodology, project administration, supervision, validation, resources, data curation.

## Ethics Statement

Clinical research permit was approved by the Department of Neurology of Helsinki University Hospital. According to the Finnish law, retrospective research using hospital medical files does not require informed consent from the study subjects. We confirm that we have read the Journal's position on issues involved in ethical publication and affirm that this work is consistent with those guidelines.

## Conflicts of Interest

Tuomas Mertsalmi has received a lecture fee from Merck. K. Amande M. Pauls has received lecture fees from AbbVie. Johanna Eerola‐Rautio has received consultation fees from NordicInfu Care. Eero Pekkonen is a member of the MDS Non‐Motor Parkinson's Disease Study Group and a consulting neurologist for the Finnish Patient Insurance Centre. He has received consulting fees from Boston Scientific, NordicInfu Care AB and AbbVie. He is a member of the advisory board in AbbVie. He has received lecture fees from AbbVie and NordicInfu Care, and is currently a PI in Finland of International Adroit‐study (Abbott DBS Registry of Outcomes for Indications over Time). 2021‐: Organized by Abbott. The other authors declare no conflicts of interest.

### Peer Review

The peer review history for this article is available at https://publons.com/publon/10.1002/brb3.70193.

## Data Availability

The data that support the findings of this study are available from the corresponding author upon reasonable request.
